# Validation of treatment decision algorithms for childhood tuberculosis at district healthcare levels in Mozambique and Zambia: the Decide TB cluster-randomised pragmatic trial – a study protocol

**DOI:** 10.1136/bmjopen-2025-114005

**Published:** 2026-05-15

**Authors:** Joanna Orne-Gliemann, Clémentine Roucher, Celso Khosa, Graeme Hoddinott, Marc d’Elbée, Minh Huyen Ton Nu Nguyet, Denise Banze, Maryline Bonnet, Katia Cossa, Emilie Desselas, Peter J Dodd, Cleia Etoa, Alan Kachuka, Natacha Lebrun, Patrick Lungu, Benedita José, Sylvia Manguele, Carla Monin, Angel Mubanga, Crimenia Mutemba, Natasha Namuziya, Laura Olbrich, Megan Palmer, Marieke M van der Zalm, James A Seddon, Chishala Chabala, Olivier Marcy, on behalf of Decide TB study group, Chishala Chabala

**Affiliations:** 1 National Institute for Health and Medical Research (Inserm) UMR 1219, Research Institute for Sustainable Development (IRD) EMR 271, University of Bordeaux, Bordeaux, Nouvelle-Aquitaine, France; 2 Instituto Nacional de Saúde, Marracuene, Mozambique; 3 Department of Physiological Science, Clinical Pharmacology, Faculty of Medicine, Eduardo Mondlane University, Maputo, Mozambique; 4 Departments of International Public Health and Clinical Sciences, Liverpool School of Tropical Medicine, Liverpool, UK; 5 School of Public Health, Faculty of Medicine and Health, The University of Sydney, Sydney, New South Wales, Australia; 6 Desmond Tutu TB Centre, Department of Paediatrics and Child Health, Faculty of Medicine and Health Sciences, Stellenbosch University, Cape Town, South Africa; 7 Department of Human, Biological & Translational Medical Science, School of Medicine, University of Namibia, Windhoek, Namibia; 8 The University of Sydney Infectious Diseases Institute (Sydney ID), Sydney, New South Wales, Australia; 9 Centre for International Health, University Hospital, LMU Munich, Munich, Germany; 10 TransVIHMI, University of Montpellier, National Institute for Health and Medical Research (Inserm), IRD, Montpellier, Provence-Alpes-Côte d’Azur, France; 11 Sheffield Centre for Health Related Research, School of Medicine & Population Health, The University of Sheffield, Sheffield, England, UK; 12 National TB and Leprosy Program, Zambia Ministry of Health, Lusaka, Zambia; 13 Department of Internal Medicine, University of Zambia School of Medicine, Lusaka, Zambia; 14 Adult’s Hospital, University of Zambia University Teaching Hospital, Lusaka, Zambia; 15 National TB Program Ministry of Health, Maputo, Mozambique; 16 Children’s Hospital, University of Zambia University Teaching Hospital, Lusaka, Zambia; 17 Institute of Infectious Diseases and Tropical Medicine, LMU University Hospital, LMU Munich, Munich, Germany; 18 German Centre for Infection Research (DZIF), Partner Site Munich, Munich, Germany; 19 Fraunhofer Institute ITMP, Immunology, Infection and Pandemic Research, Munich, Germany; 20 Department of Infectious Diseases, Imperial College London, London, UK; 21 University of Zambia, School of Medicine, Department of Paediatrics, Lusaka, Zambia; 22 University of Bordeaux, Talence, Nouvelle-Aquitaine, France

**Keywords:** Child, Tuberculosis, Clinical Decision-Making

## Abstract

**Introduction:**

Of 1.2 million children and young adolescents (<15 years) developing tuberculosis (TB) yearly, more than 50% are undiagnosed and unreported to national TB programmes (NTPs) and the World Health Organization (WHO). This is mainly due to poor performance of microbiological tests, limited clinical skills and structural barriers for childhood TB diagnosis at decentralised levels of care. Treatment decision algorithms (TDAs) could improve child TB outcomes but require external validation. We aim to evaluate a comprehensive TDA-based approach for childhood TB screening, diagnosis and treatment decision-making at district hospital (DH) and primary health centre (PHC) levels in Mozambique and Zambia.

**Methods and analysis:**

Decide TB is a pragmatic, hybrid effectiveness-implementation type 2 cluster-randomised trial with a stepped wedge design. The comprehensive TDA-based approach (intervention) will be implemented under programmatic conditions in four districts in each country (each comprising one DH and six PHCs), randomly selected to switch sequentially from the standard of care to the intervention. Evaluations will assess epidemiological, clinical, economic, social sciences, implementation and health policy endpoints. Aggregated and individual data from children with presumptive TB will be extracted from facility registers and individual data will be collected using an electronic medical record (EMR), both data sources will be entered in national Demographic Health Information System 2 databases. Questionnaires and individual/group interviews (among healthcare workers (HCWs), parents/caregivers and key informants), supervision and mentoring reports and quantitative cost tools will be used.

**Ethics and dissemination:**

Ethics approval was obtained from national ethics committees in Mozambique (Instituto Nacional de Saúde review board and National Committee for Bioethics in Health) and Zambia (University of Zambia ethical review board and National Health Research Authority); this includes a waiver for analysing data collected by NTPs (no identifiable information reported, intervention with minimal risk) without individual consent from children’s parents/caregivers. Informed consent will be obtained from HCWs, parents/caregivers and key informants. Results will be openly shared with the scientific community, WHO and national and international stakeholders for translation into policy and practice. Procedures for requesting further use of Decide TB data will be publicly available.

**Trial registration number:**

NCT06593080; PACTR202407866544155.

**STRENGTHS AND LIMITATIONS OF THIS STUDY**

- Decide TB will evaluate a comprehensive approach for screening, diagnosis and management of childhood tuberculosis (TB) using several treatment decision algorithms (TDAs) adapted to different paediatric population groups, including high-risk children. TDAs will be implemented at primary and secondary levels of healthcare, with the support of digital tools.
- Decide TB is a stepped wedge cluster-randomised trial embedded in a national TB programme-led programmatic pilot, with a hybrid effectiveness-implementation design in order to maximise transfer of research into policy and practice.
- Interdisciplinary mixed methods research will be used to assess effectiveness of TDAs, including TB detection, diagnosis accuracy of TDAs and key implementation research endpoints such as feasibility, acceptability, values and preferences, adoption, costs, cost-effectiveness and budget impact.
- The stepped-wedge design provides an opportunity to improve intervention implementation from one district to the next but at the same time limits the scope of continuous improvement of TDA implementation within each district.
- The quality of clinical data collected among children in routine conditions may be limited by the suboptimal use of digital tools due to functional and structural barriers within health facilities.

## Introduction

 Each year, an estimated 1.2 million children and young adolescents (<15 years) develop tuberculosis (TB) and nearly 250 000 die from the disease; 95% of these child deaths are estimated to result from non-diagnosis.^1^ Globally, only 44% of the estimated number of children with TB are reported to the WHO by national TB programmes (NTPs) mainly because they are not diagnosed and consequently not treated. Children with impaired immunity (eg, children living with HIV (CLHIV) or those with severe acute malnutrition (SAM)) are at higher risk of underdiagnosis and of dying from TB.^2^,^4^

The diagnosis of TB is complex in children because of challenges in collecting suitable samples for testing in younger children (who are unable to self-expectorate) and because of the poor performance of existing microbiological tests (due to the paucibacillary nature of TB disease in young children).^5^ Suboptimal TB detection is also due to structural challenges in many resource-limited countries, including (1) childhood TB services centralisation at hospital-levels and thus poorly accessible to most children, (2) lack of access to rapid molecular testing at decentralised levels of care^6^ and (3) poor access to or low quality of chest X-ray (CXR) and limited expertise in interpreting CXR images. Most children are treated for TB on the basis of clinical consideration.^7^,^11^ There is thus a critical need to strengthen the skills of healthcare workers (HCWs) in the diagnosis and treatment initiation for childhood TB^12^ and to provide them with effective health technologies, including comprehensive treatment-decision algorithms (TDAs).

TDAs are tools that assign scores to clinical, radiographic, microbiological or laboratory features and recommend TB treatment initiation above a predefined threshold.^13 14^ In March 2022, the WHO conditionally recommended the use of TDAs to diagnose pulmonary TB in children below 10 years.^15^ Of TDAs currently available, two algorithms target the general paediatric population under 10 years: Algorithm A for settings with CXR access and Algorithm B for settings without CXR,^16^ both proposed by WHO in the paediatric operational handbook that accompanied the 2022 guidelines. Two other algorithms focus on high-risk groups of children: the Paediatric Asian African Network for Tuberculosis and HIV Research (PAANTHER) TDA for CLHIV^13^ developed during the PAANTHER study and the TB Speed SAM TDA ‘one step’ for children under 5 years old hospitalised with SAM.^17^ These TDAs met the WHO-recommended sensitivity threshold of 85%, that is, limit the risk of missing children with TB: the reported sensitivities were 86% (Algorithm A), 84% (Algorithm B), 88.6% (PAANTHER) and 86.1% (TB-Speed SAM). However, specificities vary widely—37%, 30%, 61.2% and 80.9%, respectively: this raises concern about potential overdiagnosis and over-treatment and calls for current efforts in externally validating TDAs.^16^,^18^

TDAs were developed for improving children’s access to TB diagnostic services and could be a useful tool for rapid and uniform treatment decision-making within primary healthcare settings. However, there is no evidence to date on the acceptability, among end users and beneficiaries, of using such tools for childhood TB; there are no data either on their feasibility in routine implementation contexts. The use of TDAs at decentralised levels of care by HCWs with limited child TB experience will likely require the strengthening of clinical skills and treatment decision-making capacity. Digital tools such as clinical decision support systems (CDSS) available on tablets/phones could also support clinicians in managing TDA scoring systems. A CDSS would automatically attribute points to the clinical, radiological and microbiological data entered in the digital patient file and calculate the total TDA score. Although there have been a few initiatives describing the development of CDSSs in the field of TB,^19^,^21^ none have specifically addressed childhood TB diagnostics.

Apart from the PAANTHER TDA, which has been recently externally validated,^18^ the other TDAs aforementioned have not yet undergone prospective evaluation in new cohorts. WHO has called for external validation data to inform their conditional recommendation on the use of TDAs for childhood TB diagnosis; such data include data on the effectiveness, feasibility, acceptability and cost-effectiveness of TDAs, the values and preferences from end-users and TDA diagnostic accuracy.

Furthermore, based on results of the SHINE trial, which found that a 4-month regimen with the same drugs was non-inferior to the standard 6-month regimen in children with non-severe TB, WHO also recommended that a 4-month treatment regimen should be used in children and adolescents between 3 months and 16 years of age with non-severe TB.^15 22^ Although this was a strong recommendation associated with moderate certainty of evidence, the guideline identified substantial challenges in identifying children with non-severe TB, especially regarding access to CXR for disease severity assessment. Therefore, alongside the evaluation of TDAs, it is important to assess the implementation of the shorter treatment regimen for children with non-severe TB and to nest the severity assessment in a comprehensive TDA-based approach.

The general objective of the Decide TB trial is to evaluate a comprehensive TDA-based approach for childhood TB screening, diagnosis and treatment decision-making, supported by digital tools and piloted and implemented under programmatic conditions at district hospital (DH) and primary health centre (PHC) levels in Mozambique and Zambia. The intervention will integrate WHO-recommended TDAs with TDAs developed specifically for CLHIV and those with SAM, and a disease severity assessment step to assess eligibility for the shorter (4 months) treatment for children with non-severe TB. Interdisciplinary assessments will assess: (1) the effectiveness of the intervention in increasing TB case detection in children as compared with the standard of care (SOC) and its diagnostic accuracy, (2) the feasibility and implementation of the intervention, including contextual determinants of implementation, (3) its acceptability and perceived value to end-users, beneficiaries and other key stakeholders, (4) the cost of the intervention from the provider and patient’s perspective, its cost-effectiveness and the budget impact of scaling up the intervention and (5) factors that support or constrain the intervention adoption as health policy at the district level.

## Methods and analysis

### Study type/study design

**Figure 1:**
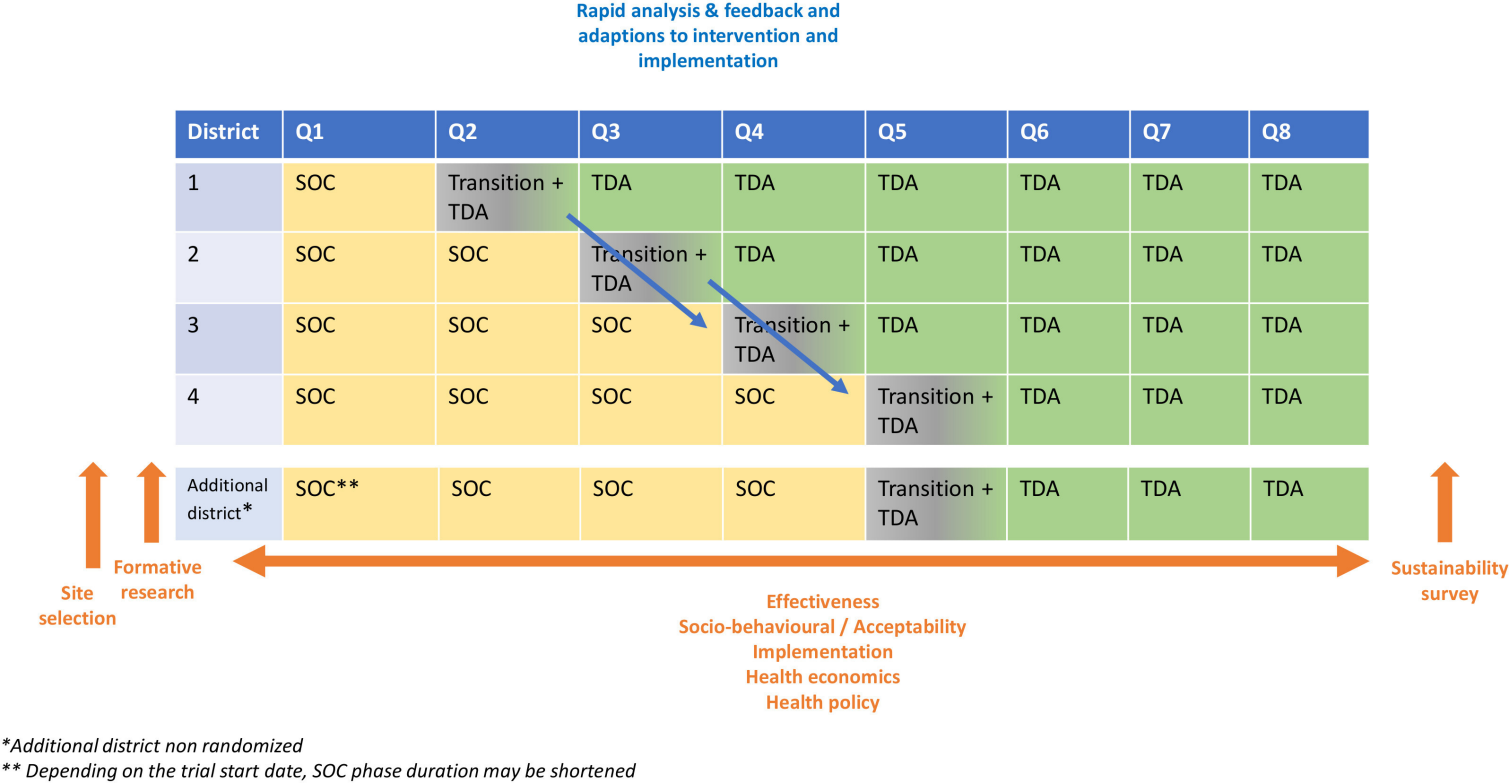
Overall study design at the country level. SOC, standard of care; TDA, treatment decision algorithm.

Decide TB is a pragmatic cluster-randomised trial (2024–2026) with a stepped wedge and hybrid effectiveness-implementation trial type 2 design. It will evaluate a comprehensive TDA-based approach (intervention) versus the SOC (control) for screening, diagnosis and treatment decision for childhood TB (see figure 1).^23^ Districts in each country will be randomly selected to successively initiate the intervention every quarter. Prior to the trial, a site selection survey will systematically identify districts and facilities that have the capacity to deploy TDAs and present a diversity of health systems and geographical characteristics.^24^ Formative research will describe the local facilities and community dynamics to highlight potential implementation barriers and facilitators and inform on intervention implementation strategies. The intervention will be implemented by the NTPs of Mozambique and Zambia. The effectiveness of the intervention in terms of TB case detection (children started on TB treatment) among children attending care will be assessed by comparing it to the SOC using both aggregated and register-based individual routine health data from the Ministry of Health (MoH). Additionally, aggregated and register-based individual data will be collected retrospectively for 12 months for secondary effectiveness endpoints (table 1). We will develop an electronic medical record (EMR) to collect individual health data among all children with presumptive TB. The EMR will be developed in Demographic Health Information System 2 (DHIS2) using the same variables for both countries. The diagnostic accuracy of the SOC and of the intervention will be assessed in an additional district in each country not participating in the stepped wedge design. The acceptability, feasibility, health economics and health policy evaluations of the intervention and of its implementation will be nested within the trial and use repeated mixed method surveys (in a convergent design). The study will start with formative research in February 2024. The stepped wedge trial will start mid-2024 and end mid-2026. The last children enrolled during the trial will be followed up until the end of 2026. A post-intervention sustainability survey will take place early 2027. Both countries will be autonomous in terms of trial start date and analysis.

**Table 1:** Data collection and tools

	Site selection and formative research	Effectiveness	Acceptability	Implementation	Health economics	Health policy	Timing of data collection *Target population*	Data collected *Data collection method*
Aggregated data	X	X					Retrospective, SOC, intervention and follow-up *Children attending care, children with TB*	Aggregated health data routinely reported to the MoH in the national DHIS2 database including OPD attendance, number of children in high-risk groups, number of TB notifications in children, among others.
Individual data-based registers		X					Retrospective, SOC, intervention and follow-up *Children with presumptive TB and TB*	Data from presumptive TB registers and from TB treatment registers *Reported in created modules in DHIS2*
EMR		X					SOC in additional district only and intervention *Children with presumptive TB*	Demographics, clinical and anthropometric data, HIV status, radiological data and abdominal ultrasound examination, laboratory data, TDA used and score, TB severity assessment, TB treatment if initiated and duration, TB treatment outcomes, comorbidity management including antiretroviral treatment, nutrition therapy and status at follow-up visit *EMR data entry on tablet in DHIS2*
Child TB questionnaire	X		X	X			Formative phase and before end of trial *HCWs*	Knowledge, attitudes and practices of HCWs, their readiness to and their perceived feasibility of TDAs for childhood TB. *Self-administered REDCap questionnaire on tablet, with support from social sciences research assistants (SSRAs*).
TB diagnosis questionnaire			X	X			Quarterly (implementation and intervention) 6 monthly (acceptability) *HCWs*	Structured in three modules: (1) implementation module, to assess the scope of childhood TB diagnosis activity, barriers and facilitators and context; (2) acceptability module, to document perceptions of appropriateness of, utility of and self-confidence towards the diagnostic approach used in the facility; (3) intervention module (intervention arm only), to document the experience of the comprehensive TDA-based approach. *Self-administered REDCap questionnaire on tablet, with support from SSRAs*
Semistructured facility observations	X		X	X		X	Six-monthly	Different aspects of TB activities will be observed: service offer, HR, patient flow, management, infrastructure, equipment and stock of supplies *Tablet-based observation checklist and notes write-up on a digital recorder*
Semistructured community observations	X		X	X			Six-monthly	Observation of access and use of healthcare services (including living conditions, road conditions, transportation), identification of key stakeholders and influential leaders *Tablet-based observation checklist and notes write-up on a digital recorder*
Usability questionnaire				X			Intervention phase Six-monthly *HCW using EMR*	HCWs’ overall satisfaction, the ease of use of tools and the learning process. If possible, this will include a qualitative ‘think aloud method’ where the participants will be asked to interact with the tool while continuously verbalising their thoughts. *Self-administered REDCap questionnaire on tablet*
Group interviews	X		X	X		X	Formative phase *Facility-in-charges/site managers, parents/caregivers*	Interview guides exploring norms and attitudes towards childhood TB diagnosis, perceptions, preferences and views towards algorithms and digital health. *Group interviews conducted face-to-face*
Individual interviews	X		X	X	X	X	Quarterly (implementation and intervention) 6 monthly (acceptability) *HCWs, district focal points, parents/caregivers, community members, civil society representatives*	Interview guides exploring attitudes towards childhood TB diagnosis, perceptions, preferences and views towards algorithms and digital health *Interviews conducted face-to-face or on the phone / via video call interviews*.
Time and motion interviews					X		SOC and intervention *HCW*	Recording of all activities related to TB patients and the length of time specific staff (nurses, doctors, radiographer/radiologist/imaging specialist and laboratory technicians) spend on each activity. *Elicited and recorded by research assistants*.
Patient cost survey					X		SOC and intervention *Parents/caregivers of children receiving treatment for tuberculosis*	Recording of costs for patient families in each country. Costs are categorised into direct medical costs, direct non-medical costs and indirect costs and are collected before and during the Decide-TB intervention. *Elicited and recorded by research assistants*.
Costing study					X		SOC and intervention *Key informants, medical, financial and administrative staff*	Recording of national and facility level costs and resource uses for the SOC and the Decide-TB intervention. *Elicited and recorded by research assistants*.
Technical support supervision and clinical mentoring reports				X		X	Monthly then quarterly	National support supervision checklists and reports revised to include TDA components, documenting strengths, challenges and improvement strategies for TDA implementation in each facility. Clinical mentoring tools include clinical mentors’ assessment of the TB diagnosis and severity and reasons for TB treatment decision diverging from score results or severity assessment results.
Sustainability survey			X	X	X	X	After the end of the trial	Quantitative and qualitative questions about reported use of TDA (HCW only), perceptions of the translation of research results into policies and practices and decision-making processes. Quantitative assessment of factors (barriers and facilitators) by HCWs. *Self-administered questionnaire by HCW on REDCap*

DHIS2, Demographic Health Information System 2; EMR, electronic medical record; HCW, healthcare worker; MoH, Ministry of Health; OPD, outpatient department; REDCap, Research Electronic Data Capture; SOC, standard of care; TB, tuberculosis; TDA, treatment decision algorithm.

### Theoretical and conceptual frameworks

**Figure 2:**
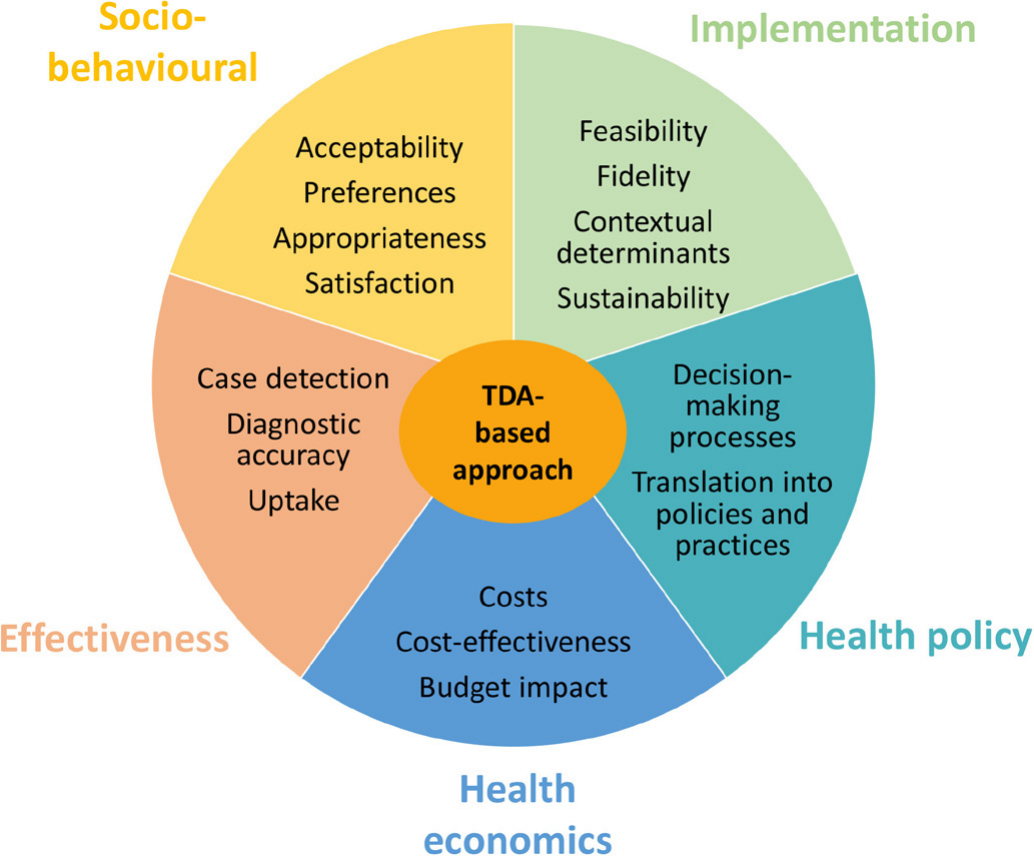
Interdisciplinary approach to the evaluation of the comprehensive TDA-based approach. TDA, treatment decision algorithm.

In order to evaluate the complex and multifaceted components of the intervention and its implementation, we will use a multidisciplinary approach combining epidemiological/clinical and sociobehavioural implementation, health economics and public policy research (see figure 2). The formative assessment will be conducted within the framework of an adapted Broad-Brush Survey methodology,^25^ supporting a pragmatic and outcome-directed approach to describing the trial communities, facilities and contexts. The comprehensive intervention and implementation evaluation will be framed in the context of the stepped wedge cluster randomised trial design in order to provide comparative evidence on all outcomes, as well as assess for time effect.^26 27^ To understand acceptability, we will apply the theoretical framework of acceptability^28^ and refer to WHO’s guidance on TB care end-user preferences.^29^,^31^ To identify the multi-level barriers and facilitators influencing intervention delivery and implementation, we will use the Consolidated Framework for Implementation Research.^32 33^ Economic evaluations of TDAs will be implementation-based, taking a complex intervention perspective and considering demand (patient costs for accessing care, preferences) and supply (availability of healthcare staff, supplies) constraints.^34^ Finally, we will also analyse stakeholder knowledge, interest, power and position in health policy implementation, clarifying how these interconnected factors shape actor influence across national, local and frontline levels.^35^

### Study settings

The study will be conducted in Mozambique and Zambia, two high TB burden countries with overall estimated TB incidences of 361 and 283 cases per 100 000, respectively.^36^ The stepped-wedge trial will be implemented in four districts in each country, each composed of one DH and six PHCs; the additional district (not randomised) will be composed of one DH and three to four PHCs. The estimated number of children attending outpatient department (OPD) in each district per quarter is 10 500, including 3500 at the DH level and 7000 children at the PHC level.

### Study populations

The effectiveness of the intervention will be assessed among: (1) all sick children below 15 years entering the selected health facilities at either OPD or inpatient departments (IPD), including children from high risk groups, as well as children identified as contact of TB cases through community-based or facility-based household contact tracing and (2) children with presumptive TB defined as per national guidelines. High-risk groups will be defined per the definition in WHO-suggested TDAs A and B (children younger than 2 years, CLHIV or children with SAM) and per country adaptations (inclusion of moderate acute malnutrition and household contacts as high risk). There will be no exclusion criteria during the programmatic pilot and trial: all presumptive TB children will be offered the intervention.

Children will be screened for presumptive TB at OPD, antiretroviral clinic, Mother and Child Health, and nutrition department when available. Children hospitalised at DH will also be screened for TB at paediatric IPD. Depending on each facility’s staffing situation, TB screening may be conducted by a cough officer/community health worker, nurse-assistant, nurse or clinical officer.

The acceptability, feasibility/implementation, health economics and health policy evaluations will be conducted among the following populations: (1) end-users (ie, HCWs involved in delivering childhood TB screening, diagnosis and treatment decision-making in the selected facilities); (2) beneficiaries (ie, parents/caregivers of sick children, as well as children willing and able to participate, identified as presumptive TB in the selected facilities); (3) key informants at district level, involved in the implementation of the intervention and supporting its delivery (ie, mainly facility-in-charge/site managers, information technology and data staff, district health teams and focal points); (4) key informants at national and international levels (such as trainers, managers, supervisors and mentors and decision/policy-makers).

### Intervention

**Figure 3:**
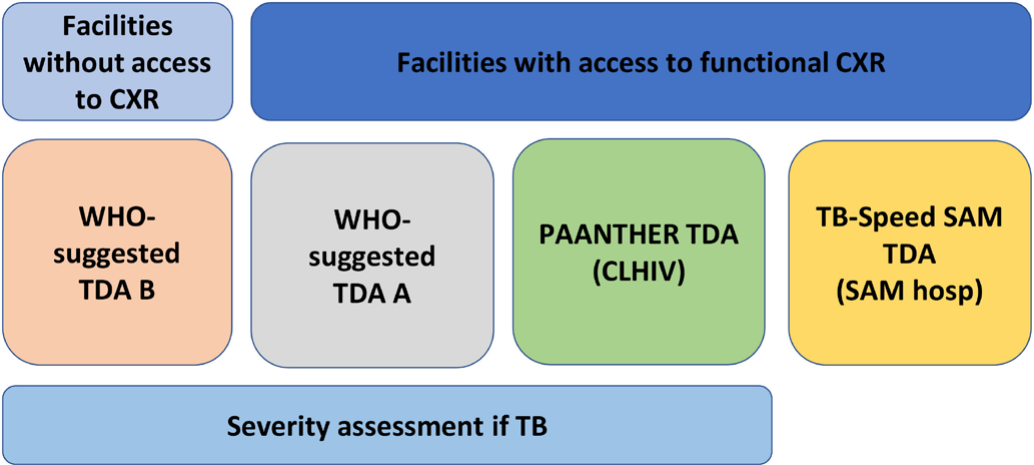
TDAs used according to entry sites and populations (PHC or DH). TB Speed SAM TDA will be used in children <5 years hospitalised with SAM. PAANTHER will be used in CLHIV<15 years. WHO TDA A or WHO TDA B will be used in children <15 years or children <10 years depending on the country adaptation. CLHIV, children living with HIV; CXR, chest X-ray; DH, district hospital; hosp, hospitalised; PHC, primary health clinic; SAM, severe acute malnutrition; TB, tuberculosis; TDA, treatment decision algorithm.

The intervention will be a comprehensive TDA-based approach for the screening, diagnosis and management of TB in children and use of shorter treatment for children with non-severe TB (see figure 3). All sick children will be assessed using WHO TDAs A (with CXR) or B (without CXR), according to CXR availability at the facility. In Mozambique children aged 10–14 years will be assessed using the national algorithm. CLHIV and children aged <5 years hospitalised with SAM will be evaluated using the PAANTHER and TB-Speed SAM TDAs, respectively, in facilities where CXR equipment is available. The components of each TDA include data on TB exposure history, clinical signs and symptoms, microbiological testing results with Xpert MTB/RIF Ultra (Ultra) on sputum, nasopharyngeal aspiration, gastric aspiration or stool, as well as CXR examination and urine lipoarabinomannan (LAM) test result in CLHIV in both countries and in children with SAM, sepsis or chronic kidney disease stage 3 or 4 in Zambia. Abdominal ultrasound will be performed at DH for CLHIV and children hospitalised with SAM, per PAANTHER and TB-Speed SAM TDAs. CXR will be performed at DH and at some PHCs, depending on the facility’s radiological capacity. Clinical and microbiological assessment data obtained throughout the use of TDAs, as well as test results, will be incorporated in a CDSS to help with the decision on TB treatment initiation and choice of TB treatment duration. This comprehensive TDA-based approach was adopted by consensus during national workshops held in July 2023 in each participating country and involving NTPs, scientific partners and the Decide TB consortium.

### SOC per country

In Mozambique, the SOC approach for child TB diagnosis relies on NTP algorithms which include: TB symptoms screening, HIV testing, Ultra testing on sputum or stool and testing with the tuberculin skin test. Urine LAM testing is indicated for CLHIV. No CXR is performed. In Zambia, the SOC follows the union’s desk guide algorithm, which involves symptom screening, HIV testing and Ultra on respiratory and stool samples. Urine LAM is more broadly used (eg, children with SAM or sepsis). CXR is performed dependent on availability. In both countries, TB diagnosis is based on symptoms, suggestive CXR or TB exposure, and TB diagnosis is confirmed if there is a positive microscopy, Ultra or LAM test result.

### Implementation strategies

Implementation strategies supporting intervention deployment will be aligned with the routine NTP implementation plans of both countries. District-level training for HCWs on TDA use, digital tool use and interpretation of imaging results will include initial face-to-face training, cascade training to other facility staff members and continuous training supported by digitalised training and support material. Monthly (first 3 months), then quarterly support supervision visits and clinical mentoring visits will be organised both on-site and online; these visits will address implementation bottlenecks and reinforce clinical skills. NTPs will lead the procurement of equipment and supplies to ensure clinicians have access to adequate sampling and testing materials and supplies for the optimal use of TDAs in the field. Continuous coordination meetings will be organised. Community mobilisation activities within facility catchment areas and districts overall will include community meetings and radio shows.

National data collection systems for childhood TB/integrated management of childhood illness will be strengthened for routine programme monitoring and for trial data collection, through the use of the DHIS2 platform on tablets. At the facility level, the EMR will be completed on tablets to report individual data for each child with presumptive TB: demographic, clinical and other data needed to calculate TDA scores and to assess disease severity. The CDSS, interconnected with the EMR module, will extract the children’s characteristics and facility profile data to orient on the choice of the TDA data and to calculate the TDA scores.

### Randomisation

In each country, health districts will be units of intervention and all will start the trial in the SOC arm. Randomisation will be done at the district level, separately for each country. For each district, the sequence will include SOC (3–12 months), intervention (3–12 months) with a lead-in/transition phase of 1 month for the stepped wedge part of the trial and then continued intervention in routine conditions with supervision (9 months) (figure 1). The randomisation list stratified by country will be generated by the trial statistician and will define the order in which each district will switch from control to intervention (every 3 months). The study coordinators will be blinded to the randomisation list and informed of the next district to switch 8 weeks prior to crossover.

### Sample size

For the effectiveness component, the statistical power to detect an increase in TB detection among children attending care (primary effectiveness endpoint) was estimated to be 0.922 for country and 0.981 for joint-analyses, using the Shiny CRT Calculator^37^ and using the following assumptions: (1) an estimated attendance of 10 500 children per quarter and per district (3000 at DH and 7500 at PHC levels); (2) a hypothesised 0.1% TB case detection rate among sick children attending care at SOC phase and 0.3% at intervention phase; (3) a discrete time decay correlation structure with a cluster auto-correlation of 0.95; (4) an intra-class correlation of 0.03 adjusted to the low prevalence of a rare binary outcome^38 39^; (4) a significance level of 5%; (5) a T-distribution applied as small sample correction for CI estimation.

Sample sizes for the acceptability, feasibility/implementation, health economics and health policy evaluations were determined as follows: (1) for HCWs (per district)—an exhaustive recruitment estimated at ~200 HCWs for the child TB and TB diagnosis questionnaires (table 1); 15 HCWs per district using digital tools for the usability questionnaire; a purposive selection of ~eight HCWs for individual interviews and of 12 HCWs per district for the time and motion study; (2) for parents/caregivers (per district): a purposive selection of 8–12 parents/caregivers for group interviews during the formative survey; of ~eight dyads (parent/caregivers-child) for individual qualitative interviews during the trial; of 240 parents/caregivers per country for patient cost surveys; (3) for key informants: a purposive selection of 5–10 stakeholders at district level and of 2–5 at provincial, national and international levels, respectively, per country.

### Endpoints

The endpoints of the different research components will be quantitative (box 1), qualitative or mixed.

Box 1 **Trial interdisciplinary quantitative endpoints.**

**Effectiveness endpoints**

- Primary endpoint: proportion of children started on treatment for tuberculosis (TB) among sick children attending care at participant health facilities for any health complaints.
- Proportion of children treated for TB among those with presumptive TB, including children with microbiologically confirmed TB.
- Proportion of children from high-risk groups treated for TB (age <2 years, children living with HIV, children with severe acute malnutrition) among all children from high-risk group attending care and among children from high-risk groups with presumptive TB.
- Proportion of children with TB who are microbiologically confirmed (ie, smear or Xpert or lipoarabinomannan positive), ratio of <5 years to 5–14 years among children with TB and ratio of pulmonary TB to extrapulmonary TB.
- Time from presumptive TB identification to final TB treatment decision and access to TB diagnostic assessment defined as the proportion of children with presumptive TB having completed assessment with the treatment decision algorithms (TDAs) and clinical decision support systemClinical Decision Support Systems, including in high-risk groups.
- Concordance of the TDA result and the final TB treatment decision using the proportion of children wrongly initiated or not initiated on TB treatment defined as: (1) children not started on TB treatment despite positive score; (2) children initiated on TB treatment despite negative score.
- Proportion of missed TB cases and over-diagnosed TB cases (unlikely TB with a positive score) compared to a reference standard diagnosis. This will be assessed in a subset of children with full diagnostic assessment (≥1 respiratory or stool sample tested with Xpert MTB/RIF Ultra, chest X-ray (CXR) availability, clinical evaluation and follow up) at district hospital or with access to CXR.
- Concordance of the severity evaluation and decision for short TB treatment regimen using the proportion of (1) children with 4-month TB treatment regimen used despite severe disease as assessed by the clinical/CXR evaluation; (2) children with 6-month TB treatment regimen despite non-severe disease as assessed by the clinical/CXR evaluation.
- Proportion of missed severe TB cases and cases over-diagnosed as severe, compared to reference standard severity assessment by expert at low level of health care: (1) children with non-severe disease according to clinical criteria despite severe disease as evaluated by expert CXR read; (2) children with clinically assessed severe disease despite non-severe disease as evaluated by expert CXR read.
- Proportion of children with presumptive TB and with non-severe TB disease initiated on shorter TB treatment.
- TB treatment outcomes as defined per WHO (treatment success, cured, treatment completion, loss to follow-up, death, treatment failure) overall and stratified by severity and regimen duration.
- Deaths averted assessed through modelling.
- Number of adults with TB, including microbiologically confirmed TB, and proportion of all TB diagnosed that is child TB.

**Acceptability endpoints**

- Co-primary endpoints: clinicians stating that in their facility they are confident in their ability to decide to initiate TB treatment in a child <5 years/in a child 5–14 years.
- Secondary endpoint: clinicians stating that in their facility they are confident in their ability to decide to initiate TB treatment in a child.
- Secondary endpoints: clinicians stating that in their facility they are not over-diagnosing children <5 years/5–14 years/all children, that is, initiating children on TB treatment when they actually do not have TB.
- Secondary endpoints: clinicians stating that in their facility they are supporting children <5 years/5–14 years/all children with TB to access care/medication as swiftly as they should.

**Feasibility and implementation endpoints**

- Co-primary endpoint: healthcare workers (HCWs) stating that in their facility they have the adequate tools to diagnose children <5 years for TB/5–14 years for TB*.
- Secondary endpoint: HCWs stating that in their facility they have the adequate tools to diagnose any children (<15 years) for TB*.
- Secondary endpoint: clinicians stating that in the last 3 months they started a child on TB treatment.

**Health economics endpoints**

a. Cost analyses of the standard of care (SOC) and of the comprehensive TDA-based approach

- Total TB care costs from the health system perspective.
- Unit cost per TB care activity (eg, clinical assessment, CXR).
- Total TB care costs of overdiagnosis (false positive)—intervention only.
- Total costs incurred by parents/caregivers for receiving child TB care.

b. Cost-effectiveness of the comprehensive TDA-based approach compared to the SOC:

- Modelled health impact measure:
  - Number of children treated for TB.
  - Number of adults treated for TB.
  - Number of deaths.
  - Disability-adjusted life years (DALYs): measure of healthy life lost, either through premature death or living with disability due to illness.
- Cost-effectiveness measure, that is, incremental cost per:
  - Incremental number of children treated for TB.
  - Death averted.
  - DALY averted.

c. Budget impact of the comprehensive TDA-based approach scale-up compared to the SOC over 5 years:

- Cost of scaling up the comprehensive TDA-based approach nationally.
- Cumulative number of children and adults treated for TB, lives saved and DALYs averted during a 5-year scale-up.

The primary effectiveness endpoint will be the proportion of children started on TB treatment among sick children attending care at selected facilities for any health complaint, calculated during the SOC and intervention periods. Secondary effectiveness endpoints include the calculation of other proportions (eg, proportion of children treated for TB—including with TB confirmed or at high risk for TB—among all children with presumptive TB, among all children from high-risk groups attending care, etc), disaggregated by age and TB status. Time between presumptive TB identification and treatment decision and initiation will be measured. Concordance between TDA results and TB treatment decision and between severity evaluation and short treatment regimen decision will be calculated. Diagnostic accuracy (sensitivity, specificity, negative and positive predictive values) of the TDAs and severity assessment accuracy will be assessed based on a reference standard determined by an endpoint review committee taking into consideration diagnostic and follow-up data.

The acceptability of the intervention will be reported as the preferences of HCWs in delivering childhood TB diagnosis and care (comparing the intervention vs SOC), the perceived social value of the intervention for HCWs and beneficiaries and the associated health systems and socioeconomic and contextual factors influencing overall acceptability.

The feasibility/implementation endpoints will be the feasibility of delivering and implementing childhood TB diagnosis and care and specifically the intervention; the fidelity to intervention delivery plan and adherence to implementation guidelines; the adaptations in implementation over time; the contextual factors influencing implementation and the sustained delivery of the intervention 6 months post implementation.

Health economics endpoints will include total TB care costs from both health system and patient perspectives and estimated unit costs per care activity. The cost-effectiveness of the TDA-based approach will be evaluated through incremental cost per children with TB diagnosed and treated, alongside incremental costs per death and disability-adjusted life years (DALYs) averted. Additionally, a 5-year budget impact analysis will estimate national implementation costs and endpoints such as cumulative cases treated, lives saved and DALYs averted.

Finally, health policy endpoints will be the roles and practices among implementers and decision-makers at the district level regarding the intervention, their perceived usefulness of the intervention, their option of the intervention and the mechanisms for translating research findings into public policies and practices.

### Data collection and tools

For the effectiveness component, aggregated data originally obtained from facility registers and entered in the national DHIS2 database will be extracted; individual data from registers (TB treatment and TB presumptive registers) will also be entered on specific DHIS2 modules, in addition to the EMR. For the analysis of endpoints collected at the intervention phase and the diagnostic accuracy assessment, we will use the individual patient data collected in the EMR. For the acceptability, feasibility/implementation and policy components, we will use both quantitative data (child TB questionnaire, TB diagnosis questionnaire) and qualitative data (interviews, supervision and clinical mentoring reports, field notes). The health economics component will use questionnaires. Quantitative data from the acceptability, feasibility/implementation, health economics and policy components will be entered on the electronic data capture application Research Electronic Data Capture (REDCap; https://www.project-redcap.org/). Interviews will be captured on audio-recording devices (with respondent consent) and electronically typed/transcribed. Table 1 details the data collected, tools and methods contributing to the research components and endpoints.

### Data management and monitoring

All individual data, clinical or collected through questionnaires and interviews will be coded using numerical identification codes. Individual and aggregated data routinely collected on DHIS2 will be stored on the servers hosted by the country’s MoH. Data collected with the EMRs will be transferred from DHIS2 to the Decide TB research database and stored on a secure data repository hosted by the University of Bordeaux; this transfer will be done using RENATER’s (French national telecommunications and data network for research and education) secure file transfer system with individual authentication. The data will be pseudonymised before transfer from the NTPs. The other research data collected (eg, audio recordings, reports, qualitative field notes) will be stored on a secure data repository via a secure file transfer protocol (SFTP) at the University of Bordeaux. The research databases on DHIS2 and REDCap will be accessible by secure authentication to a restricted user’s group. The connection will be authenticated by a user ID, password and digital certificate enabling data encryption during transfer and storage to the central server. Implementation data, such as supervision and mentoring reports stored at MoH, will be anonymised and transferred on the SFTP.

Data routinely collected on DHIS2 by NTP will be monitored using the monitoring and evaluation system in place at the country level, with additional support from the in-country and international research teams. A data monitoring committee is not needed because we are in a programmatic pilot using routine data. Quality control of quantitative and qualitative research data will include checks to identify inconsistent, incomplete or inaccurate data.

### Data analysis

The analysis of quantitative effectiveness, acceptability and feasibility endpoints will be described in the statistical analysis plan written by the trial statistician and approved by the trial scientific advisory board (SAB). A specific health economics analysis plan will be written.

#### Effectiveness endpoints

For the effectiveness endpoints, data will be analysed both at trial (joint analysis) and country levels (country analysis). The primary effectiveness analysis will focus on the stepped-wedge period (five quarters) and compare all endpoints using aggregated or individual register-based data between SOC and intervention. Generalised estimating equations^40^ with fixed effects for steps and intervention will be used. Data from the transition period will be removed. We will conclude that the intervention is more effective than the control if the observed difference in outcome is statistically significant (p<0.05). Model goodness-of-fit will be assessed, and alternative solutions will be discussed and considered, if relevant. Sensitivity analysis will use the same approach but will consider data from the transition period as intervention data. Secondary analysis will be planned and performed on all districts to (1) assess TDA-based approach effectiveness on diagnostic accuracy and implementation and (2) account for secular trend and potential bias of the study effect. Subgroup analyses will be performed considering the type of vulnerability (including age group), sex, facility-level and country. Analyses will be carried out with R V.4.4 or higher.

#### Acceptability, feasibility/implementation and health policy endpoints

Primary and secondary quantitative acceptability and feasibility endpoints (see table 1) will be analysed through the same stepped wedge trial analysis as for the primary effectiveness endpoint.

Rapid analysis methods will be used for the qualitative data collected right before and after the switch from control to intervention: the aim is to provide rapid feedback to implementers and key stakeholders at local and national levels and inform adaptation of implementation strategies to context. This analysis will be based on daily debriefs between social science research assistants and investigators, completion of a thematic matrix and summary of findings per implementation components. For the in-depth analysis of the qualitative data, a coding framework will be developed, based on themes predefined by the semistructured interview guides and emerging from the data as responsive to the acceptability, feasibility/implementation and health policy endpoints. Thematic summaries will be prepared for each endpoint, highlighting contextual specificities. Analyses will first be conducted country by country separately and then merged (and compared/contrasted when relevant) during finding consolidation and interpretation. Finally, quantitative and qualitative data will be combined and triangulated to assess certain acceptability, feasibility/implementation and health policy endpoints.

#### Health economics endpoints

Mean costs between arms will be calculated and reported descriptively alongside their 95% uncertainty intervals but will not be compared statistically. Cost analyses will be conducted following WHO guidelines.^41^ Total and unit cost per child will be examined by country and by subgroups such as CLHIV and/or SAM, type of cost inputs, type of TB care activity and by type of health facility (DH vs PHC). Household cost analysis will be conducted following WHO guidelines.^42^ The within-trial cost-effectiveness analysis will use the effectiveness endpoints (proportion of children started on TB treatment among sick children attending care, including children from high-risk groups). The cost and effectiveness results from the model will then be combined to calculate incremental cost-effectiveness ratios which will be compared with the countries’ cost-effectiveness thresholds. Once diagnostic accuracy data become available, further economic modelling will generate projections for mortality and morbidity, calculated based on DALYs, which will be used to calculate the expected overall health outcomes for patients following each alternative arm. The budget impact analysis will estimate the additional costs and savings of implementing the intervention compared with current health spending for the SOC and apply these costs to the whole population. The scale-up costs will include the upfront cost of new equipment and training as well as the running costs of TB diagnosis and treatment services.

### Patient and public involvement

The protocol was reviewed by the CISPOC Community Advisory Board in Mozambique and the SAB includes one community representative who was involved in discussing and approving the trial protocol.

## Ethics, oversight and dissemination

### Ethical approvals

The Decide TB protocol will be conducted in accordance with the applicable laws and regulations in Mozambique and Zambia, the principles stated in the Declaration of Helsinki and the Good Clinical Practice guidelines. Instituto Nacional da Saúde (INS) and University of Zambia (UNZA) are the sponsors of the study and had a role in designing the study and in collecting the data. The trial protocol V.3.0 dated 20 August 2025 was approved in Zambia (UNZA ethical review board—reference number 4292–2023 and National Health Research Authority—reference number HREB001/24/11/2023) and in Mozambique (INS review board reference number 005/CIE-INS/2024 and National Committee for Bioethics in Health—reference number 77/CNBS/2024). All amendments to the protocol will be communicated with the relevant parties including sponsors and ethics review boards. The protocol is registered in clinicaltrials.gov (NCT06593080) and in PanAfrican Clinical Trial Register (PACTR202407866544155).

### Scientific advisory board

An independent SAB will review protocol amendments, progress and results and ensure scientific and ethical integrity of the trial. SAB members include technical experts on childhood TB, stepped wedge randomised trials and implementation research; representatives from WHO, UNICEF, the TB Union and NTPs from Cameroon and Uganda; end-users/HCWs and representatives from the community. The SAB will have access to interim analyses. In addition, each country will set up a country project committee which includes key country stakeholders including community representatives.

### Consent

Individual child data within the Decide TB trial will be collected routinely by the NTPs in national registers and the national DHIS2 database. As no identifiable information will be reported and the intervention poses minimal risk to children, a waiver was granted from Mozambique and Zambia national research ethics committees and no individual consent is needed from parents/caregivers for the analysis of the data. HCWs, parents/caregivers and key informants will be provided an information sheet and consent form, adapted to each population group, and will only be consented once to participate in the trial mixed methods research activities (see online supplemental material). Participants to the interviews will be reimbursed for the travel expenses incurred to take part in the interview and will be offered healthy refreshments.

### Dissemination

Results from the Decide TB trial will be rapidly and openly shared with the scientific community, WHO and national and international stakeholders (including other sub-Saharan African NTPs) for implementation and translation into policy and practice. Trial findings will be presented at district (participating facilities) and national levels in Zambia and Mozambique. Plain language summaries will be developed for community dissemination. Study results will be published in peer-reviewed journals and presented at national and international conferences. All publications will be agreed on by coordinating investigators, the SAB and sponsors and will respect the international recommendations.^43^ Primary peer-reviewed articles will be published in ‘gold’ or ‘green’ open access journals. Other dissemination (workshops, symposia, conferences, policy briefs, etc) and external communication activities (website, leaflets, community engagement, etc) will be conducted to ensure broad visibility and engagement with the trial findings and outcomes.

Decide TB data will be used, after the publication of the main study results, for secondary studies and country-specific analyses. It may be used outside of the consortium for contributions to individual data meta-analyses, additional secondary analysis or analyses unrelated to the Decide TB trial topic. Data access will be granted on the basis of a clear justification of the use of data (concept note) and on agreement from the trial executive committee/investigators, as per Decide TB publication and access to data charter’s dispositions.

## Discussion

The Decide TB trial is conducting a hybrid evaluation of TDAs for childhood TB screening, diagnosis and treatment decision-making in order to inform both effectiveness and implementation outcomes. The implementation of TDAs as a programme pilot by NTP Mozambique and Zambia, together with interdisciplinary evaluation, will ensure comprehensive external validation of those diagnostic tools as called for by WHO and improve generalisability of findings.

The Decide TB trial will provide a package of evidence on the effectiveness in increasing TB case detection and diagnostic accuracy of TDAs, their feasibility, acceptability by end-users, effectiveness, costs and cost-effectiveness. The identification of barriers and facilitators for implementation and conditions for better implementation of these interventions will be key for implementers and policymakers to improve the quality of TB service delivery and scale-up of interventions. The evidence provided will be used to update clinical curricula, to inform national policies and to update the current WHO policy/childhood TB guidelines and operational handbook related to the use of TDAs for pulmonary TB diagnosis in children. Decide TB will thus contribute to filling up the detection gap of childhood TB and improving childhood mortality and morbidity.

## Supplementary material

10.1136/bmjopen-2025-114005 online supplemental file 1
