# Supplementary material for: Validation of treatment decision algorithms for childhood tuberculosis at district healthcare levels in Mozambique and Zambia: the Decide TB cluster-randomised pragmatic trial – a study protocol
Source: BMJ Open. 2026 May 15;16(5):e114005. doi: 10.1136/bmjopen-2025-114005 (PMC13182456; doi:10.1136/bmjopen-2025-114005)
Supplement: online supplemental file 1 [file bmjopen-16-5-s001.docx]

PARTICIPANT INFORMATION LEAFLET AND CONSENT FORM

| **Title of THE STUDY** | |
| --- | --- |
| **VALIDATION OF TREATMENT DECISION ALGORITHMS FOR CHILDHOOD TUBERCULOSIS AT DISTRICT HEALTH CARE LEVELS IN MOZAMBIQUE AND ZAMBIA – THE DECIDE-TB CLUSTER-RANDOMIZED PRAGMATIC TRIAL (THE DECIDE-TB TRIAL)**  **SOCIAL SCIENCE COMPONENT OF THE TRIAL / HEALTH CARE WORKERS** | |
| **DETAILS OF PRINCIPAL INVESTIGATOR (PI):** | |
| **Title, first name, surname: XXX** | **Ethics reference number: XXX** |
| **Full postal address: XXX** | **PI Contact number: XXX** |

We would like to invite you to take part in the social science component of the Decide-TB trial. Please take some time to read the information presented here, which will explain the details of this social science component of the trial. Please feel free to ask the research assistant, who handed you this form, any questions about any part of this social science component of the trial that you do not fully understand. It is very important that you are completely satisfied and that you clearly understand what this research entails and how you could be involved. You may also contact the primary investigator of the Decide-TB trial, at any point in time (see contact details below). Your participation is **entirely voluntary** and you are free to decline to participate. Refusal to participate will involve no penalty or loss of benefits to which you are otherwise entitled. You are also free to withdraw from the social science component of the trial at any point, even if you do agree to take part initially.

The xxx Ethics Committee have approved the Decide-TB trial and this social science component (xxx). The social science component of the trial will be conducted according to the ethical guidelines and principles of the international Declaration of Helsinki, xxx (refer to any national guidelines or standards).

**What is this research all about?**

- Decide-TB is a trial evaluating the use of Treatment Decision Algorithms to improve the diagnosis of childhood tuberculosis in Mozambique and Zambia.
- Decide-TB is being carried out by the National Tuberculosis Programs of Mozambique and Zambia in collaboration with University of Zambia, Institute of Health of Mozambique, University of Bordeaux and the University of Stellenbosch, among others.
- Over the 2-year course of the trial, 5 districts in each country will be trained and supported to implement Treatment Decision Algorithms for childhood TB. This will happen in sequence: after a few months of the trial, 1 district will be randomly selected to start using Treatment Decision Algorithms, then 3 months later another district will be randomly selected to start using Treatment Decision Algorithms, etc.
- Within the Decide-TB trial, we will conduct social science research, that aims to document how key people understand and experience the Treatment Decision Algorithms: these are Health Care Workers (HCWs), parents, community members, and staff involved in the implementation, management and decision-making around childhood TB. We also wish to describe precisely the time it takes for HCWs to deliver the Treatment Decision Algorithms and the costs for the health system.
- Within this social science component of the trial, we wish to conduct several research activities that involve HCWs from the trial sites. More specifically, we will invite:
  - all HCWs in the trial sites in Zambia and in Mozambique to respond to a questionnaire on childhood TB, that will take place before and after the trial;
  - all HCWs in the trial sites in Zambia and in Mozambique to respond to a questionnaire on TB diagnosis, that will be repeated every 3 months during the social science component of the trial, and one last time a few months after the end of the trial;
  - a small number of HCWs to take part in qualitative interviews (group discussions/focus groups, and also individual interviews). We will use a technique called “purposive sampling”, in order to interview HCWs that have specific socio-demographic or professional characteristics. That means that even if you, as a HCW, agree to participate in the interviews, only a very small number will be considered “eligible” and will be formally invited to participate;
  - all HCWs in the trial sites in Zambia and in Mozambique to take part in anonymous observations (to assess the time taken by different activities), before and during the social science component of the trial.

**Why are you invited to participate?**

- As a HCW we would like to invite you to participate in this research so that we can take into account your personal experience, opinion and views in the improvements made for childhood TB diagnosis in Mozambique and Zambia.

**What will your responsibilities be?**

- For the childhood TB questionnaire, you will:
  - Receive a tablet and respond to a brief questionnaire on what you know and think about childhood TB. It may take you between 10-20 minutes to complete it; this varies according to people. There are no right or wrong answers and all information will be kept confidential.
  - Respond to this questionnaire once before the start of the trial, and once at the end of the trial.
- For the TB diagnosis questionnaire, you will:
  - Receive a tablet to respond to a brief questionnaire capturing your thoughts and feelings about childhood TB diagnosis. It may take you between 10-20 minutes to complete it; this varies according to people. There are no right or wrong answers and all information will be kept confidential.
  - Respond to this questionnaire every 3 months.
- For the qualitative interviews (both group and individual), you will:
  - Attend a meeting with our trained social sciences research assistant, which will consist in an individual interview that will take between 30 minutes to 1 hour.
  - Attend such meeting every 6 months.
- For the time and motion study, you will:
  - Spend time with a research assistant who will anonymously record all activities related to TB care and the length of time that you spend on each activity. These observations are not aimed at "controlling" the quality of care provided, but only to better understand the time needed for you to provide TB care.

**Will you benefit from taking part in this research?**

- There are no direct advantages to your participation, but by participating and sharing your experiences you are contributing to a social science component of the trial that can potentially improve TB care in children.

**How the information you have shared (my data) will be protected?**

- All data collected will be handled with confidentially and no names will be used neither included in reports. We will replace your name with a unique participant identifier. They are said to be pseudonymized or coded.
- Once the research is completed, we must store your data for 15 years. This will be done in a secured database located in France for storage and analysis. The sponsors of the project (UNZA/INS) and University of Bordeaux are responsible for this treatment and for the protection of your personal data.
- You have the right to access your data or to request for it to be deleted from the database at any time. You also have a right of rectification, a right to refuse or to limit the use of your data.

**What will be done with your data?**

- When the social science component of the trial is finished, we would like to publish results in scientific journals. Most journals require us to share coded individual data with them before they publish the results. Therefore, we would like to obtain your permission to have your coded individual data shared with journals.

For research purposes, other investigators working on TB or other infectious diseases, can also ask to use this information or part of it in the future. If this happens, it will be done according to signed agreement and approval between the investigators and under security rules. **Re-use of your data for further research**

- Your data may be further processed in connection with the same or other infectious diseases and in a context other than that described in the current protocol. You have the option of accepting or refusing the principle of this re-use in the consent form provided to you.

**Are there any risks involved in your taking part in this research?**

- There are minimal risks with you taking part in this social science component of the trial.
- We estimated that taking part in all activities of this social science component of the trial would take 25mn to 1h30 of your time, every 3 months.

**Are there any costs involved if you decide to participate/take part?**

- You will be not compensated for your time in taking part in any of the activities of the social science component of the trial, however refreshments will be provided and any travel costs will be covered. You will not have to pay for anything related to the research if you do take part.

**Is there anything else that you should know or do?**

- You can phone [insert PI’s name here] at [insert PI’s telephone number here] if you have any further queries or encounter any problems.
- You can phone the xxx Ethics Committee at xxx if there still is something that the researcher has not explained to you, or if you have a complaint.
- You will receive a copy of this information and consent form for you to keep safe.

**Declaration by participant**

By signing below, I ……………………………………………. agree to take part in the social science component of the Decide-TB trial, “Validation of treatment decision algorithms for childhood tuberculosis at district health care levels in Mozambique and Zambia – the Decide-TB cluster-randomized pragmatic trial”.

I declare that:

- I have read this information and consent form, or it was read to me, and it is written in a language in which I am fluent and with which I am comfortable.
- I have had a chance to ask questions and I am satisfied that all my questions have been answered.
- I understand that taking part in this social science component of the trial is **voluntary,** and I have not been pressurized to take part.
- I understand that accepting to take part in this social science component of the trial does not mean that I will take part in all research activities (because for some activities the researchers will sample from all HCWs who accept to participate)
- I may choose to refuse some of the activities included in the social science component of the trial activities, or leave the social science component of the trial at any time and nothing bad will come of it – I will not be penalized or prejudiced in any way.

I understand that my coded data might be shared with scientific journals and/or with other investigators

I agree to take part in the social science component of the trial of the Decide-TB trial, “Validation of treatment decision algorithms for childhood tuberculosis at district health care levels in Mozambique and Zambia – the Decide-TB cluster-randomized pragmatic trial”.

□ Yes □ No

I agree to have my data collected during the study used for further research on TB or other infectious diseases in a coded way.

□ Yes □ No

Name of the participant ......................…........……………........................…........……………..

Signed at (place) ......................…........…………….. on (date) …………....………...

Signature of participant

**Declaration by investigator**

I (name) …………………………………………… declare that:

- I explained the information in this document in a simple and clear manner to …………………………………..
- I encouraged him/her to ask questions and took enough time to answer them.
- I am satisfied that he/she completely understands all aspects of the research, as discussed above.
- I did/did not use an interpreter. (If an interpreter is used then the interpreter must sign the declaration below.)

Signed at (place) ......................…........……………... on (date) …………....………...

Signature of investigator
