# Supplementary material for: Validation of treatment decision algorithms for childhood tuberculosis at district healthcare levels in Mozambique and Zambia: the Decide TB cluster-randomised pragmatic trial – a study protocol
Source: BMJ Open. 2026 May 15;16(5):e114005. doi: 10.1136/bmjopen-2025-114005 (PMC13182456; doi:10.1136/bmjopen-2025-114005)
Supplement: Supplementary file 2 [file bmjopen-2025-114005.reviewer_comments.pdf]

PEER REVIEW HISTORY

BMJ Open publishes all reviews undertaken for accepted manuscripts. Reviewers are asked to complete a checklist review form and are provided with free text boxes to elaborate on their assessment. These free text comments are reproduced below.

ARTICLE DETAILS

Title (Provisional)

Validation of treatment decision algorithms for childhood tuberculosis at district health care levels in Mozambique and Zambia - the Decide TB cluster-randomized pragmatic trial: a study protocol

Authors

Orne-Gliemann, Joanna; Roucher, Clementine; Khosa, Celso; Hoddinott, Graeme; d'Elbée, Marc; Huyen Ton Ngu Nguyet, Minh; Banze, Denise; Bonnet, Maryline; Cossa, Katia; Desselas, Emilie; Dodd, PJ; Etoa, Cleia; Kachuka, Alan; Lebrun, Natacha; Lungu, Patrick; José, Benedita; Manguele, Sylvia; Monin, Carla; Mubanga, Angel; Mutemba, Crimenia; Namuziya, Natasha; Olbrich, Laura; Palmer, Megan; van der Zalm, Marieke M; Seddon, James A; Chabala, Chishala; Marcy, Olivier; Decide TB study group, on behalf of

---

VERSION 1 - REVIEW

**Reviewer**	**1**
**Name**	**Moon, Troy**
**Affiliation**	**Tulane University**
**Date**	**22-Jan-2026**
**COI**	**None**

This is a valuable and interesting piece of work which when completed will address important gaps related to WHO recommendations for TDAs in this population. However, as is the protocol leaves open a lot of questions that need further clarification.

Major:

- Line 13 pg 8, please better describe what a CDSS is, as to clarify its difference from a TDA? How do they work together? What information will one have that the other doesn't? Also are you developing the CDSS for this study or is it already existing?
- Line 25, Pg 10. Districts in both Mozambique and Zambia can have quite different geographies, patient populations, and health infrastructure that impacts on the number of quarterly outpatient visits at each facility and within

each district. How did you come up with the singular number of outpatient visits that will occur in each district per quarter?

- Line 34 pg 10, for your effectiveness evaluation are you including all children as your denominator or only those suspected to have TB
- Line 35 Pg 10. It's not clear if you are including both outpatient and inpatients as your denominator for evaluation. If including inpatient numbers, please explain why? Especially since your patient estimates Line 25-29 Pg 10 refer to outpatient only.
- In the Study population section, I would recommend providing more detail as to all portals of entry into the study (Where screening will take place) and by whom you think screening will be performed.
- In the intervention section and Figure 3, it is not clear if more than one TDA will be utilized at a given health facility. What happens if a child with HIV or SAM is seen at a PHC. Much greater detail as to which TDA will be utilized and in which facilities is needed.
- Lines 10-20 Pg 12: It is not clear if the study will be developing an EMR or using EMR's already in existence. Should this actually refer to a study database rather than an EMR? For example the REDCap database referred to at Line 30 Pg 14. If you are referring to a true EMR , somewhere you should describe the differences and similarities across countries as to the data collected and whether that is harmonized for this study?
- The authors need to be consistent throughout the text as to what the different endpoint measures are. For example Line 43 Pg 12, it is stated the primary effectiveness endpoint is TB detection, whereas Line 23 Pg 13, it is proportion of children started on treatment. Table 1 does not mention TB detection as a primary endpoint. Then the Discussion section Line 16 Pg 18 states the study will provide evidence as to the effectiveness of increasing detection and diagnostic accuracy and does not mention TB treatment.
- In the data analysis section Pg 15, please describe whether there will be any comparisons made between the different TDA's or if the TDA's are grouped as a unit and then compared to SOC. This is not clear.
- Consider added a Study limitations section

Minor:

- Conduct a review and ensure all numbers use a decimal point rather than a comma. For example, the first sentence of Abstract is 1,2 million rather than 1.2 million
- Review text and be consistent, ensuring that all whole numbers that are less than ten are spelled out, rather than using the number itself.
- Please conduct a review of the entire text. Authors have a tendency to use run-on sentences that reduce clarity and readability. For example, the first sentence

of the introduction could be broken into two sentences between the words "disease" and "95%".

- Line 27 pg 7, its not clear what you mean when you say "low quality of chest X-ray".
- Line 49 pg 7, spell out PAANTHER the first time it is used
- Line 48 pg 8, please edit "Te intervention"
- Line 57, pg 9, please clarify what SW-CRT is referring to and verify that all abbreviations are spelled out the first time used.

VERSION 1 - AUTHOR RESPONSE

Reviewer: 1

Prof. Troy Moon, Tulane University

Comments to the Author:
This is a valuable and interesting piece of work which when completed will address important gaps related to WHO recommendations for TDAs in this population. However, as is the protocol leaves open a lot of questions that need further clarification.

Reviewer: 1
If you have selected 'Yes' above, please provide details of any competing interests.: Not applicable

Major:
• Line 13 pg 8, please better describe what a CDSS is, as to clarify its difference from a TDA? How do they work together? What information will one have that the other doesn't? Also are you developing the CDSS for this study or is it already existing?

Response: A clinical decision support system (CDSS) is a health information technology system designed to assist health care professionals in clinical decision-making; it can be implemented as a computer-based tool, web platform or a mobile application, and may rely on rule-based algorithms or in some cases artificial intelligence techniques. A Treatment Decision Algorithm (TDA) is not a health information technology system per se, it's an approach with a set of rules that guide the clinician in managing a patient from one step to the next, here from TB presumption to TB treatment decision. Throughout the TDA algorithmic pathway, points are assigned to the patient's health data, whether clinical and radiographic features or microbiological test results, and a total score is calculated. Above a pre-defined score threshold, the TDA recommends TB treatment initiation. In Decide TB, we are using an electronic medical record (EMR) to record all the patient's health data, and an embedded CDSS that automatically attributes the points and calculates the TDA score.

Revised text: P.8, Lines 7-12: "Digital tools such as Clinical Decision Support Systems (CDSS) available on tablets/phones, could also support clinicians in managing TDA scoring systems. *A CDSS would automatically attribute points to the clinical, radiological and microbiological data entered in the digital patient file and calculate the total TDA score. Although there have been a few initiatives describing the development of CDSSs in the field of TB, none have specifically addressed childhood TB diagnostics*."

• Line 25, Pg 10. Districts in both Mozambique and Zambia can have quite different geographies, patient populations, and health infrastructure that impacts on the number of quarterly outpatient

visits at each facility and within each district. How did you come up with the singular number of outpatient visits that will occur in each district per quarter?

Response: Our estimation of the quarterly outpatient attendance among children (7,500 at DH and 3,000 at PHC) in Mozambique and Zambia is based on the TB Speed Decentralisation study experience. Within this study, conducted in 2021-2022 in 5 sub-Saharan African countries (Mozambique, Cameroon, Uganda, Sierra Leone, Côte d'Ivoire) and in Cambodia, the median quarterly outpatient attendance among children > 15 years was 10,214 at DH and 2,828 at PHC (https://doi.org/10.1016/j.eclinm.2024.102527). Although we did not implement the TB-Speed study in Zambia, estimates for OPD attendance were similar and we were aiming for districts with such numbers.

• Line 34 pg 10, for your effectiveness evaluation are you including all children as your denominator or only those suspected to have TB

Response: As specified P.10, Lines 29-34, the effectiveness of the comprehensive TDA-based approach will be assessed using multiple endpoints with a strong focus on case detection. The primary endpoint will be the proportion of children detected with TB among all sick children below 15 years entering the selected health facilities at either outpatient departments (OPD) or inpatient departments (IPD). Secondary effectiveness endpoints will include the number of children detected with TB among ii) children with presumptive TB defined as per national guidelines, iii) children from high-risk groups.

• Line 35 Pg 10. It's not clear if you are including both outpatient and inpatients as your denominator for evaluation. If including inpatient numbers, please explain why? Especially since your patient estimates Line 25-29 Pg 10 refer to outpatient only.

Response: We will include primarily outpatient children for our effectiveness assessment, taking into consideration the fact that most inpatient children are usually counted as entering as outpatients. This is in line with our previous work in the TB-Speed Decentralization study (https://doi.org/10.1016/j.eclinm.2024.102527). Children attending inpatient department will be screened for TB at District Hospital, especially children hospitalized with severe acute malnutrition will be assessed with the TB Speed SAM TDA. However, our estimated attendance was only based on children entering outpatient department and main results will be based on that.

• In the Study population section, I would recommend providing more detail as to all portals of entry into the study (Where screening will take place) and by whom you think screening will be performed.

Response: Staff will be trained to conduct TB screening at all entry points in the facility, and by which ever staff is available that day (whether volunteers, nursing or clinical staff).

Revised text: P.11, Lines 1-4: *Children will be screened for presumptive TB at OPD, ART (antiretroviral) clinic, MCH (Mother and Childhood Health), and nutrition department when available. Children hospitalized at DH will also be screened for TB at paediatric IPD. Depending on each facility's staffing situation, TB screening may be conducted by a cough officer/community health worker, nurse-assistant, nurse or clinical officer*.

• In the intervention section and Figure 3, it is not clear if more than one TDA will be utilized at a given health facility. What happens if a child with HIV or SAM is seen at a PHC. Much greater detail as to which TDA will be utilized and in which facilities is needed.

Response: In facilities without access to CXR equipment, i.e., most PHCs, all presumptive TB children will be assessed with the WHO-suggested TDA B. In facilities with access to CXR equipment, presumptive TB children with be assessed with the WHO-suggested TDA A; if they are living with HIV or less than five years and hospitalized with SAM, they will be evaluated using the PAANTHER and TB-Speed SAM TDAs, respectively. We have clarified the use of TDAs for different populations and in different facilities in the manuscript accordingly.

Revised text: P.11, Lines 18-22: *All sick children will be assessed using WHO TDAs A (with CXR) or B (without CXR), according to CXR availability at the facility. CLHIV and children less than five years hospitalized with SAM will be evaluated using the PAANTHER and TB-Speed SAM TDAs respectively, in facilities where CXR equipment is available*.

Revised Figure 3:

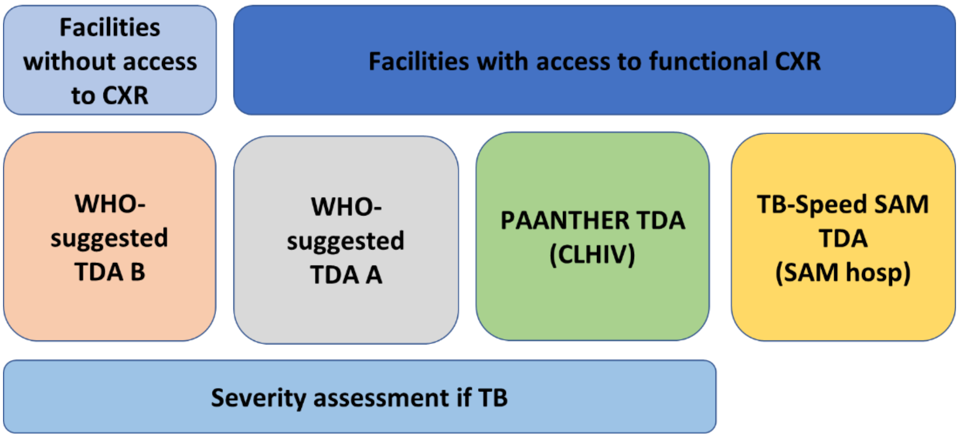

• Lines 10-20 Pg 12: It is not clear if the study will be developing an EMR or using EMR's already in existence. Should this actually refer to a study database rather than an EMR? For exemple the REDCap database referred to at Line 30 Pg 14. If you are referring to a true EMR, somewhere you should describe the differences and similarities across countries as to the data collected and whether that is harmonized for this study?

Response: Both the Mozambican and Zambian Ministries of Health (MoH) are using the DHIS2 platform (District Health Information Software) to manage district and facility health data; they have also been developing digital information systems for individual patient data recording and management (such as SmartCare in Zambia, or SIS-TB in Mozambique). In coordination with both countries' National TB Programmes, it was decided that the study would develop a specific patient Electronic Medical Record (EMR) to collect individual health data among children with presumptive TB. The Decide TB EMR has been developed using the tracker module of the DHIS2 system, already in use in both countries. Data collected in the DHIS2 EMR will be stored on the servers hosted by the country MoH. Data will be transferred from DHIS2 to the Decide-TB research database in Bordeaux, France, and stored on a secure data repository hosted by the University of Bordeaux.
The REDCap database referred to P.14, lines 33-35 will be newly developed to collect the quantitative data from the acceptability, feasibility/implementation, policy and health economics components. We have clarified this.

Revised text:

- Study type/Study design P.9, Lines 24-26: *We will develop an electronic medical record (EMR) to collect individual health data among all children with presumptive TB. The EMR will be developed in DHIS2 using the same variables for both countries.*

- Data collection and tools P.14, lines 33-35: *Quantitative data from the acceptability, feasibility/implementation, health economics and policy components will be entered on the electronic data capture application REDCap (Research Electronic Data Capture; https://www.project-redcap.org/).*"

• The authors need to be consistent throughout the text as to what the different endpoint measures are. For example, Line 43 Pg 12, it is stated the primary effectiveness endpoint is TB detection, whereas Line 23 Pg 13, it is proportion of children started on treatment. Table 1 does not mention TB detection as a primary endpoint. Then the Discussion section Line 16 Pg 18 states the study will provide evidence as to the effectiveness of increasing detection and diagnostic accuracy and does not mention TB treatment.

Response: Our primary effectiveness endpoint is the proportion of children started on treatment for TB among sick children attending care at participant health facilities for any health complaints. This endpoint corresponds to TB case detection in sick children overall. Indeed, in NTPs, TB cases are notified to the WHO on the basis of the TB treatment register (i.e. TB treatment initiation = TB case detection). If some patients are diagnosed with TB and not initiated on treatment for various reason, they do not appear as being notified hence not detected.

Revised text: P.9, Lines 20-23 in the Study type / study design section: *The effectiveness of the intervention in terms of TB case detection (children started on TB treatment) among children attending care will be assessed by comparing it to the SOC, using both aggregated and register-based individual routine health data from the Ministry of Health (MoH).*

• In the data analysis section Pg 15, please describe whether there will be any comparisons made between the different TDA's or if the TDA's are grouped as a unit and then compared to SOC. This is not clear.

Response: There will be no comparison between the different TDAs. The comprehensive TDA-based approach including the use of all TDAs, i.e. the intervention, will be compared to the SOC overall.

• Consider added a Study limitations section

Response: We have described study limitations in the "Strengths and limitations of this study" section. Limitations listed include: i) the stepped-wedge design offers opportunity to improve intervention implementation from one district to the other but may reduce the ability to measure and implement continuous improvement of TDA implementation within each district ; ii) the quality of clinical data collected among children may be limited by the sub-optimal use of digital tools in routine conditions, due to functional and structural barriers within health facilities.

Minor:

• Conduct a review and ensure all numbers use a decimal point rather than a comma. For example, the first sentence of Abstract is 1,2 million rather than 1.2 million

Response: This has been corrected.

• Review text and be consistent, ensuring that all whole numbers that are less than ten are spelled out, rather than using the number itself.

Response: This has been corrected.

• Please conduct a review of the entire text. Authors have a tendency to use run-on sentences that reduce clarity and readability. For example, the first sentence of the introduction could be broken into two sentences between the words “disease” and “95%”.

Response: We acknowledge this, and have tried to cut down the length of our sentences as much as possible.

• Line 27 pg 7, its not clear what you mean when you say “low quality of chest Xray”.

Response: CXR raises 3 types of issue: i) low access as it is often only at hospital level and not always functional, ii) poor quality (due to specific challenges in paediatric CXR notably in obtaining a good CXR with appropriate rotation, penetration, inspiration and without defective lung field), and iii) lack of reading skills.

Revised text: P.7, Lines 13-16: *“Sub-optimal TB detection is also due to structural challenges in many resource-limited countries, including i) childhood TB services centralization at hospital-levels and thus poorly accessible to most children, ii) lack of access to rapid molecular testing at lower levels of care (6) and iii) poor access to or low quality of chest X-ray (CXR) and limited expertise in interpreting CXR images.”*

• Line 49 pg 7, spell out PAANTHER the first time it is used

Response: This was spelled out.

• Line 48 pg 8, please edit “Te intervention

Response: This has been corrected.

Line 57, pg 9, please clarify what SW-CRT is referring to and verify that all abbreviations are spelled out the first time used.

Response: SW-CRT is the abbreviation for stepped wedge cluster-randomized trial. We opted for not using the abbreviated form.
